# Zinc Signals and Immunity

**DOI:** 10.3390/ijms18102222

**Published:** 2017-10-24

**Authors:** Martina Maywald, Inga Wessels, Lothar Rink

**Affiliations:** Institute of Immunology, RWTH Aachen University Hospital, Pauwelsstr. 30, 52074 Aachen, Germany; martina.maywald@rwth-aachen.de (M.M.); iwessels@ukaachen.de (I.W.)

**Keywords:** zinc flux, zinc wave, homeostatic zinc signal, signaling pathways, innate and adaptive immunity, zinc deficiency, immune function

## Abstract

Zinc homeostasis is crucial for an adequate function of the immune system. Zinc deficiency as well as zinc excess result in severe disturbances in immune cell numbers and activities, which can result in increased susceptibility to infections and development of especially inflammatory diseases. This review focuses on the role of zinc in regulating intracellular signaling pathways in innate as well as adaptive immune cells. Main underlying molecular mechanisms and targets affected by altered zinc homeostasis, including kinases, caspases, phosphatases, and phosphodiesterases, will be highlighted in this article. In addition, the interplay of zinc homeostasis and the redox metabolism in affecting intracellular signaling will be emphasized. Key signaling pathways will be described in detail for the different cell types of the immune system. In this, effects of fast zinc flux, taking place within a few seconds to minutes will be distinguish from slower types of zinc signals, also designated as “zinc waves”, and late homeostatic zinc signals regarding prolonged changes in intracellular zinc.

## 1. Introduction

The metal zinc is nowadays well established to be essential for a well-operating immune system. However, knowledge about zinc homeostasis, zinc deficiency, and related diseases is comparatively new. In 1963, Dr. Prasad proved for the first time the existence of zinc deficiency in man [1]. Since then, knowledge about zinc evolved rapidly uncovering molecular mechanisms being indispensable for regulating zinc homeostasis in humans. Its significance as a structural component in proteins [2] and its participation in numerous cellular functions include, but are not limited to, cell proliferation and differentiation [3,4], RNA and DNA synthesis [5,6], stabilization of cell structures/membrane [7,8], as well as redox regulation [9,10], and apoptosis [11,12]. Zinc is involved in various metabolic and chronic diseases such as: type 1 diabetes, rheumatoid arthritis, cancer, neurodegenerative diseases, and depression [13,14,15,16,17,18,19]. Moreover, there is also strong evidence between zinc deficiency and several infectious diseases such as shigellosis, acute cutaneous leishmaniosis, malaria, human immunodeficiency virus (HIV), tuberculosis, measles, and pneumonia [20,21].

When zinc deficiency was first discovered, it was thought to be a rare disease. However, zinc deficiency is very common, with estimated two billion people worldwide being affected, and is identified as a major contributor to the burden of disease in developing countries. It is the 5th leading life-threatening factor, especially in developing countries [22]. In addition, industrial counties are affected by zinc deficiency, particularly the elderly population [23]. Despite zinc deficiency and related symptoms can easily be treated by proper zinc intake, suboptimal zinc status cannot simply diagnosed by reason of the lack of clinical signs and reliable biochemical indicators of zinc status. To date, no specific and reliable biomarker of zinc status is known, although serum/plasma zinc concentrations, hair zinc concentration, and urinary zinc excretion can be seen as potentially useful. Nevertheless, zinc status is highly impacted by the immune status itself (infection, inflammatory conditions), but also by diet, absorption and conserving mechanisms via gastrointestinal tract and kidneys [24]. Zinc uptake in the gastro intestinal (GI) tract is facilitated by an influx into the enterocyte, through the basolateral membrane and the transport into the portal circulation. Uptake mechanisms are not fully understood yet, however zinc transporters are mainly involved in zinc uptake or zinc efflux [25]. In this regard, Zrt-like, Irt-like protein (ZIP)4 is highly important since it is expressed along the entire GI tract acting as a major processor of zinc uptake into enterocytes from the apical membrane [26]. Moreover, zinc transporter (ZnT)3, is highly expressed in the human large and porcine small intestine and the esophagus [27,28]. Herein, its concrete function in the GI tract is largely unknown. However, studies in the esophagus uncovered its co-localization with sensory neuromediators and/or neuromodulators that are essential for the control of all functions of the GI tract either under physiological and pathological conditions as well as during diseases [27,28,29]. Hence, there is an ongoing need for the discovery of a reliable biological marker of zinc status.

Although the plasma pool is very small, it is highly important for cellular signaling since it is rapidly exchangeable and mobile. Consequently, intracellular zinc level can be altered resulting in altered cell function and differentiation [30,31]. The zinc-dependent regulation of the immune system is particularly interesting and will be discussed in more detail in this review. We will particularly focus on the importance of different types of zinc signals in innate as well as adaptive immunity, and highlight altered signaling pathways due to changed intracellular free zinc level.

## 2. Zinc Homeostasis and the Immune System: An Overview

With a total amount of 2–4 g, zinc is the second most abundant trace metal in the human body, iron being first. In contrast to the latter, zinc cannot be stored and has to be taken up via food daily to guarantee sufficient supply. A large number of especially inflammatory diseases, but also aging, pregnancy, lactation, and vegetarian or vegan lifestyles are associated with zinc deficiency. Thus, an important role of zinc in development and exacerbation of diseases can be assumed, as indicated earlier. This underlines the importance of a deep understanding of the various functions of zinc in the immune system and thereby for health and disease [21,32,33,34]. Already marginal-to-moderate zinc deficiency impairs immunity, delays wound healing, causes inflammation-independent low-grade production of inflammatory cytokines and increases oxidative stress [35]. Immune cells might even react more quickly to zinc deficiency than it is measurable in the plasma [36]. Imbalances including strong zinc deficiency but also zinc overload cause severe immune dysfunctions. Zinc intoxication is however rare and its symptoms mostly due to copper deficiency. Immunological hallmarks of zinc deficiency are thymic atrophy, lymphopenia, especially decreased CD4^+^ T helper (Th) cell numbers, resulting in a decreased CD4^+^/CD8^+^ ratio [37]. In vitro data suggest that monopoiesis is increased during zinc deficiency [38], natural killer (NK) cell activity is decreased and monocyte cytotoxicity is increased [21]. Zinc dependent alterations in chemotaxis, phagocytosis, respiratory burst and formation of neutrophil extracellular traps by innate immune cells offer one explanation for the increased susceptibility to infections during zinc deficiency [39,40,41].

In humans, high zinc concentrations are found in retina (3.8 µg/g dry weight), choroid of the eye (274 µg/g) and in bone (100–250 µg/g), while only 1 µg/mL zinc is found in plasma, which equals around 0.1% of total body zinc. Within body fluids, zinc is predominantly bound to proteins including albumin, α2 macroglobulin (A2M), transferrin and others. Hypoalbuminemia can even result in zinc deficiency [42]. Zinc binding to those proteins can activate or inactivate their activity, or change characteristics important for substrate binding [43].

Zinc homeostasis is primarily controlled via the expression and action of 14 zinc transporters that increase cytoplasmic zinc (Zip1–14) and 10 zinc transporters that lower cytoplasmic zinc (ZnT1–10). Zrt-like, Irt-like proteins (Zip) are also named solute carrier family 39 (SLC39) A1–A14, while members of the zinc transporter (ZnT) family are also denoted SLC30A1–A10. Many of those transporters are found spanning the plasma membrane, but others are also located within mitochondrial, Golgi network, lysosomal, and vesicular or endoplasmic reticulum membranes. This implies that decreasing cytoplasmic zinc can describe export via ZnTs, but also the transport of zinc into one of those organelles [44]. The significance of zinc transportation via nicotinic acetylcholine receptors, voltage dependent calcium channels, transient receptor potential channels and glutamatergic receptors as well as of facilitated diffusion of zinc bound to amino acids compared to specific zinc transport through Zips and ZnTs remains to be defined in more detail [45]. Together, those zinc transporters regulate zinc homeostasis on tissue but also single cell and even intracellular level.

Physiological changes in body zinc homeostasis during acute phase response include the temporal transfer of serum zinc to the tissues, especially the liver, causing transient serum hypozincemia, which is rebalanced during resolution of the inflammatory response. The transient change in zinc homeostasis is proposed to act as a danger signal for immune cells [46]. In addition, pro-inflammatory acute phase proteins including interleukin-6 (IL-6) upregulate expression of zinc binding peptides such as metallothionein (MT) and A2M, augmenting zinc sequestration from extracellular microorganisms [43]. Intracellularly increased zinc can intoxicate engulfed pathogens and acts cytoprotective by promotion of neutralizing reactive oxygen and nitrogen species (ROS and NOS) as will be discussed later. Undernourishment and severe inflammatory diseases are paralleled with prolonged and severe forms of serum hypozincemia. An association of excessively elevated levels of inflammatory markers, reactive oxygen species and antimicrobial peptides such as calprotectin or matrix metalloproteases (MMP) that cause tissue injury especially in lung, liver and spleen with the augmented serum hypozincemia was suggested [46,47,48,49].

Measuring free intracellular zinc levels results in extremely low numbers in the pico- to nanomolar range, depending on the cell type. When zinc is transported into the cell, it is efficiently buffered by high affinity proteins. As affinity of zinc to metal binding sites of proteins is rather high and competitive towards other metal ions, free zinc concentrations fluctuate in a narrow range. Excess zinc ions are transported into subcellular stores such as the including interleukin (ER) or Golgi or into extracellular space [2]. Intracellular zinc-binding proteins include members of the MT family. If in addition to their strong binding activity to extracellular zinc, members of the S100 family can also chelate zinc intracellular is so contested [50]. As the decrease in intracellular zinc during monopoiesis is paralleled by an increase in S100A8 and S100A9 expression but not changes were found for MT-1 levels, binding of intracellular zinc by calprotectin in myeloid cells is likely [38].

MTs are proteins of 6–7 kDA, which can bind and quickly release up to 7 zinc ions, thereby sequestering up to 20% of intracellular zinc. Furthermore, they protect against various types of environmental stress, as MTs can chelate other heavy metals, decreasing their cytotoxicity and they can scavenge reactive oxygen species. Within the four MT classes, MT-1 and MT-2 are expressed ubiquitous throughout the body, whereas MT-3 and MT-4 are expressed cell type specific [44]. Within innate immune cells, MT is an important molecule for zinc regulation. MT deficiency impairs cytokine production and anti-microbial activities in macrophages after lipopolysaccharide (LPS) stimulation [51]. The S100 protein family includes 24 members, all acting calcium dependent and having calcium buffering capacities. Intracellular, they modulate apoptosis, transcription and enzyme activities; extracellular S100 proteins regulate chemotaxis and wound healing, but also proliferation and differentiation via surface receptors [52]. Zinc binding S100 proteins include S100B, S100A1, S100A2, S100A3, S100A5, S100A7, S100A8/9, S100A12 and S100A16. Calprotectin, a heterodimer of S100A8 and S100A9, is the most abundant protein of neutrophils. Increased plasma levels are found during severe inflammatory diseases paralleling serum hypozincemia, suggesting a link that has recently been explored [53,54]. Some low molecular binding partners of zinc have been found as well including adenosine triphosphate (ATP), glutathione, citrate, nicotinamine or bacillithiol [2].

## 3. Classification of Zinc Signals

Signaling cascades are highly complex and very sensitive to altered intracellular second messenger concentrations. Since zinc is established to act as second messenger comparable to calcium [55], it is obvious that cellular signals are altered due to changed intracellular zinc concentrations. Regarding this, free intracellular zinc level influence signaling pathways by binding reversibly to regulatory sites in signaling proteins, altering protein activity and stability [56]. Moreover, alterations can result from the transport of zinc through the plasma membrane, exchange with intracellular organelles or zinc binding proteins as MT and calprotectin, or a combination of these mechanisms.

Intracellular zinc signals can be differently classified. One way is by the time scale they occur, comprising: (1) a “zinc flux” occurring within seconds to minutes; (2) slightly slower zinc signals, described as “zinc wave” occurring within several minutes; and (3) homeostatic zinc signals occurring within several hours. Examples for each classification of zinc signals occurring in different cell types and signaling pathways are summarized in Table 1.

A zinc flux can arise by triggering receptors such as Toll like receptor (TLR)4 in, e.g., monocytes. In this connection, zinc acts as second messenger, comparable to calcium, by influencing signaling cascades in a direct manner. This kind of zinc signal is independent of synthesis of the zinc transporter proteins from the ZnT and Zip family responsible for zinc re-distribution and zinc uptake and is therefore classified as zinc flux. Activation of a zinc transporter to enable a sharp rise in cytoplasmic zinc is thus possible.

Second, zinc signals described as “zinc wave” were uncovered recently. Comparable to the zinc flux, zinc waves act also as second messenger, but are induced indirectly depending on calcium influx. This phenomenon was for instance observed in mast cells, when the Fc-epsilon receptor I (FcεRI) is cross-linked [55,57].

Late zinc signals occur on a timescale significantly longer than the others, accompany cellular differentiation, and last for several days. Those are usually involved in altered expression of proteins participating in zinc homeostasis. In this regard, maturation of for instance monocytes and dendritic cells (DC) as well as cytokine expression is highly dependent on zinc signals [38,58]. In general, altered zinc homeostasis results in changes in signaling cascades without acting as second messenger.

## 4. Zinc, Signaling and Immunity

In addition to its function as second messenger, another suggested mechanism how zinc is involved in signaling is that zinc alters ligand binding to receptors either by changing the ligand’s or the receptor’s affinity. Here, LPS revealed altered fluidity depending on zinc’s availability and thereby binding characteristics to receptors such as TLR4 and CD14 were affected as well as subsequent signal transduction [89].

Another possible scenario is the alteration of cellular membrane composition and fluidity [90]. Here, zinc could influence the generation and stability of membrane complexes causing assembly or disassembly of receptors and probably altering their endocytosis, as is well described for neuronal cells, but has also been observed in immune cells [84,91]. Furthermore, alterations of extracellular zinc conditions affect the concentration of free intracellular zinc so that persistent changes in extracellular zinc modify cell metabolism on the long run. Recent data indicate, that there is a major difference in the effects of zinc on signaling in innate compared to adaptive immune cells. Therefore, data will be discussed separately.

### 4.1. Innate Immune Cell Functions

Cells of the innate immune system, including principally neutrophil granulocytes and monocytes/macrophages, but also mast cells, DCs, and NK cells are the first to encounter invading pathogens at the side of infection. Recognition of pathogens and initiation of their clearance needs to be fast, which is only possible if specificity is compromised. Efficient immune response is completed by adaptive immune cells, which need longer to be recruited and activated, but are highly specific.

**Figure 1:**
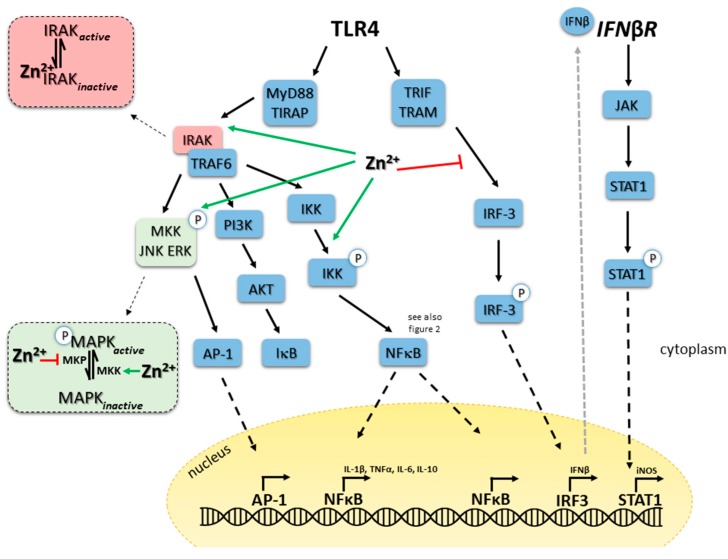
Zinc in TLR4 triggered signaling, illustrating explanations in the text. Black arrow: activation, green arrow: activating function of zinc, red T bar: inhibiting function of zinc, black dotted arrow, translocation of the molecule to nucleus, grey dotted arrow: secretion of the molecule. Abbreviations: ERK: extracellular Signal-regulated Kinase; IFN: interferon; IRAK: Interleukin-1 receptor-associated *kinase*; IκB: Inhibitor of NFκB; IKK: IκB kinase; IRF: interferon related factor; JAK: JNK janus kinase; JNK: c-Jun N-terminal Kinase; MAPK: mitogen activated protein kinases MEK: MAPK/Erk kinase; MKK: MAPK kinase; MKP: MAPK phosphatase; MyD88: Myeloid differentiation primary response gene 88; NFκB: nuclear factor (NF) κB. PI3K: phosphatidyl-inositol-3-phosphate; STAT: signal transducers and activators of transcription; TBK: Tank-binding kinase 1; TIRAP: toll-interleukin 1 receptor (TIR) domain containing adaptor protein; TLR: toll like receptor; TRAF: TNF receptor-associated factor; TRAM: TRIF-related adaptor molecule; TRIF: Toll-interleukin-1 receptor (TIR) domain-containing adaptor-inducing interferon.

Innate immune cells recognize pathogens via detection of general pathogen-associated molecular patterns (PAMPs). Receptors are simply denoted pattern recognition receptors (PRRs) and examples include TLR, retinoic acid-inducible gene-I-like receptors (RLR) and nucleotide-binding oligomerization domain-like receptors (NLR). At least 10 different TLRs exist in humans, enabling cells to distinguish groups of pathogens and their intracellular or extracellular location. Lipoprotein (gram positive bacteria), zymosan (fungi) and LPS (gram negative bacteria) are for example detected by TLR1, -2 and -4, respectively [92]. Binding of a PAMP activates signaling pathways leading to antimicrobial processes including cytokine production, degranulation, phagocytosis of the pathogens and the presentation of the antigen to other cells, including those of the adaptive immune system. Ligation of PRRs generally leads to activation of interleukin-1 receptor-associated kinase (IRAK) family proteins through Myeloid differentiation primary response gene (MyD)88 signaling pathways as depicted in Figure 1 and can either induce gene expression via nuclear factor kappa B (NFκB) or mitogen activated protein kinases (MAPK). Binding to NLRs directly leads to activation of TAK1, NFκB and MAP kinase pathways [93]. Of note, most cells are equipped with the identical sets of signaling molecules, but choice of signaling pathway(s) activated depends on the type of pathogens, so that appropriate reaction to attack the invader is induced. First data connecting zinc-induced changes in immune cell function to regulation of intracellular signaling by zinc arose in the late 1970s [94,95]. Direct effects of zinc on signaling molecules or indirect effects via phosphatases, kinases and redox metabolism have been described since then.

### 4.2. Zinc and (De-)Phosphorylation

In the following, we will first discuss some general mechanisms of how zinc affects intracellular signaling molecules and their activity in innate immune cells. Subsequently, we will use this information to describe selected key receptor-induced signaling pathways for the different innate immune cell types. This is far from being inclusive for all the pathways investigated so far, but should give a basic idea of the state of research. One should carefully extrapolate known mechanisms within certain pathways from one cell type to another, over simplifying those mechanisms as other parameters such as environmental circumstances, activation status, maturity grade and age of a cell have been shown to matter as well.

In general, signal transduction is predominantly regulated by post-translational activation or inactivation of existing signaling molecules via changes in their phosphorylation status. Each signaling pathway involves phosphorylation of a whole cascade of molecules, where phosphorylation and thereby the signal can be passed on from one molecule to the other within a short period of time. Various residues within the molecules can be altered, but tyrosine and to a lesser extend serine and threonine phosphorylation are most common in immune cells. In leukocytes, especially Src family kinases are prevalent. As phosphorylation can be both at activating and inhibitory motifs, there is not a general consequence of elevated kinase activity. Phosphorylation by kinases can be removed by antagonizing phosphatases, balancing the extent of phosphorylation inside the cells. CD45, a transmembrane protein tyrosine phosphatase (PTPs) found generally on all leukocytes, is for example an important regulator of Src family kinase activity [65,73]. Zinc has a role in regulation of PTP and PT kinase (PTK) activity. Of note, kinase and phosphatase activity is not only affected by intracellular zinc homeostasis, but both are involved in changing expression of genes relevant for regulation of zinc homeostasis, as well.

Over 107 PTPs have been identified in humans. They are primarily regulated via the redox state of the cell, proteolysis, dimerization and phosphorylation. The IC_50_ for zinc of important PTPs involved in immune cell signaling are summarized in Table 2. The table clearly shows that inhibition constants for PTPs are very low (nanomolar), so that cellular zinc levels are sufficient to alter enzymatic activity [96]. PTP1B, for example, has a putative affinity for inhibitory zinc as low as 3–17 nM [10,97]. Zinc competes with other co-factors of PTPs, which might explain their inhibition during high zinc conditions [98,99]. In addition, zinc directly acts on the highly conserved catalytic region of PTPs, suggesting that other enzymes from this family might be inhibited by zinc in a similar way [98,100]. SHP-2, phosphatase and tensin homolog deleted on chromosome 10 (PTEN) and PTP1B are discussed to be involved in LPS-induced signaling pathways in macrophages via different pathways and molecules [5,101]. This is in line with the observation that zinc prolonged the tyrosine phosphorylation of proteins in many immune pathways [55,99,102], involving all kinds of signaling molecules, underlining the complexity of zinc´s effects.

In addition to PTPs, zinc is able to inhibit the activities of other molecules involved in signaling. However, most results were calculated from experiments using isolated enzymes or cell lysates. The resulting IC50 concentrations for example for PDEs in the µM range will hardly be reached within the cytosol of intact cells. However, a few studies indicate that high levels of zinc can be enriched in cellular compartments such as vesicles and lysosomes. Hennigar et al. found 20× higher zinc concentrations normalized to protein comparing cytosol and lysosomes [103]. Moreover, in catalytically active lysosomes the non-bound Zn^2+^ concentration was measured to be close to the permissive concentration of 0.1 μM. In inactive vesicles at neutral pH the Zn^2+^ concentration might be much higher as the driving force of the proton gradient is missing [104]. In addition, Vinkenborg et al. stated that free zinc(II) concentrations in vesicles are much higher than in the cytosol, due to different buffering capacities. Estimates are 1–100 μmol/L in insulin-storing granules for example [105]. More investigations on intracellular effects of zinc on activity of those signaling molecules are necessary.

Close relatives to PTPs, the MAPK phosphatases (MKP), can dephosphorylate threonine residues in addition to tyrosine in MAPK and might be zinc’s target as well. The zinc flux has indeed been connected to inhibition of MKPs in neuronal cells, which should be tested for immune cells as well [61].

As zinc-induced secretion of cytokines by peripheral blood mononuclear cells (PBMCs) is abolished if cells are pre-incubated with the kinase inhibitor Herbimycin A, zinc might not only affect PTPs but also their antagonists, the tyrosine kinases (TK). Zinc-associated activation of tyrosine kinases has been noted by Vener et al., in cells from Alzheimer patients [106]. Results from Bennasroune et al. support this hypothesis, describing that zinc activates anaplastic lymphoma kinase (ALK), also a member of the receptor tyrosine kinase family [107]. Zinc was found within the molecular structure of other TKs including Bruton’s (B)TK as well [108]. Here, zinc is probably involved in enzymatic activity or substrate binding. BTK is an important regulator of for example TLR4 signaling via Myeloid differentiation primary response gene (MyD)88 and also binds to TLR9, -8, and -6 [109]. Activation of mitochondrial Src tyrosine kinase by zinc indicates that effects of zinc are not limited to the cytoplasm [110].

Well-known substrates for Tyrosine (de-)phosphorylation are the MAPKs, such as MAPK/Erk kinase (MEK), Extracellular Signal-regulated Kinase (ERK), and p38, which are themselves involved in phosphorylation of other signaling molecules. A role of zinc in alteration of signal transducers and activators of transcription (STAT) phosphorylation has recently started to emerge [111,112]. Effects of zinc on MAPK activity have been described for all kinds of cells, and seem to be a very general molecular mechanism to translate the zinc signal into cellular function. PTP1 is known to alter the phosphorylation of JAK2 and dephosphorylates MAPKs in immune cells [97,113]. High zinc concentrations reduced phosphorylation of ERK in in rat glioma cells, while low zinc concentrations activated this MAPK [98]. Along with this p38 was activated in myeloid cells during zinc deficiency [48]. In contrast, zinc activated ERK in fibroblasts. Similar effects of severe zinc deficiency and high levels of zinc have been observed before, and might explain the variation of results. In addition, there might be a certain threshold of intracellular zinc concentrations decisive for its influence on signaling pathways.

### 4.3. Zinc and Redox Metabolism: Two Strongly Intertwined Second Messengers

Balancing the redox state of a cell is important for regulation of its metabolism. Amongst others, ROS function as second messengers and interfere thereby with cellular signaling. Phorbol 12-myristate 13-acetate (PMA) activates the nicotinamide adenine dinucleotide phosphate (NADPH) oxidase via protein kinase C (PKC) resulting in ROS production. PMA is also a well-known inducer of a zinc flux [122]. As NADPH oxidase inhibition blocked the PMA-induced zinc signal, the release of zinc into the cytoplasm seems to be ROS-induced. H_2_O_2_ was shown to increase intracellular zinc in granulocytic cells as well, underlining the association of zinc homeostasis with redox metabolism. Both the zinc signal and the increased ROS levels are essential to subsequently activate key immune functions [40]. ROS can induce release of zinc from proteins such as MTs or PKC itself, but the source could also be a cellular compartment such as lysosomes as shown for T cells or the endoplasmatic reticulum [65,102,123,124,125]. The source of zinc probably depends on cell type, magnitude of ROS alteration and the kind of oxygen species (O_2_^−^, H_2_O_2_, HOCl or similar).

Interestingly, not only does the redox metabolism influence intracellular zinc homeostasis, but the zinc status of a cell also alters its redox state [126]. Pro-antioxidant functions have long been attributed to zinc for decades, and underlying mechanisms for this phenotypic observation are becoming increasingly clear. Although low concentrations of a zinc chelator TPEN (*N*,*N*,*N*′,*N*′-Tetrakis(2-pyridylmethyl)ethylenediamine), 5 µM) were not able to affect PKC activity, using 100 µM of TPEN inhibited PKC’s function and thereby ROS synthesis. The abrogation of PKC activation when zinc is chelated is suggested to be due to the requirements of zinc finger structures for PKC’s activity, but also because translocation of the enzyme between membrane and cytoskeleton involves zinc [122,127,128]. MAPK phosphatase (MKP)-1 is possibly also involved in LPS-induced cytokine induction via PKC. As MKP-1 is directly inhibited by zinc, the role of the trace metal in regulating LPS-induced effects seems to be complex and involve several levels, PKC being rather upstream of other effects. In contrast, long lasting zinc deficiency increased basal ROS levels and augmented stimulation induced ROS synthesis in myeloid cells [48] underlining again the difference between long- and short-term zinc alterations. Regarding signaling in innate cells, analyses of the role of zinc in oxidative burst added nice data to the puzzle, but more data are still necessary as some discrepancies exist [40,41]. An increase of ROS production by granulocytes during zinc deficiency seems to be clearly documented, whereas the effect of high zinc conditions has not been clearly defined, yet [40,41,48,129,130,131].

### 4.4. Zinc, Cyclic Nucleotides and Proteinases A and G

The intracellular second messengers cyclic adenosine monophosphate (cAMP) and cyclic guanosine monophosphate (cGMP) are synthesized by adenylate cyclase (AC) and guanylate cyclase (GC). Both are degraded by cyclic nucleotide phosphodiesterases (PDE). Their main targets are protein kinase A (PKA) and PKG, respectively, mediating inflammatory gene expression. On the one hand, PDEs need zinc for their activity, as zinc is tightly bound to the catalytic center [118,120]. This was first observed in PDEs in Baker’s yeast, indicating that this is a conserved mechanism [132]. On the other hand, PDEs have an inhibitory site, which binds zinc with lower affinity. If zinc is available in high concentrations, hydrolysis of cAMP and cGMP is inhibited, causing their intracellular increase. In human monocytes, PDE 1, 3 and 4 have been shown to be inhibited during high zinc conditions. Furthermore, zinc seems to be involved in PDE expression as well, blocking LPS-induced increase in PDE4B transcription in monocytes, adding one more way of how zinc regulates intracellular second messenger levels and thereby signaling [60]. Finally, elevated cGMP does not only target PKG but also cross activates PKA. Subsequently, PKA phosphorylates Raf-1 at serine 259, rendering it inactive and preventing its activation by serine 338 phosphorylation. Thereby the whole cascade of LPS-induced NFκB signaling pathway is suppressed [133]. Cyclic nucleotides are not only affected by zinc flux, but the homeostatic zinc signal has been shown to inhibit AC, causing decreased cAMP levels, while GC activity and cGMP levels are not affected [134,135]. Increased cAMP and cGMP levels have been associated with monopoiesis, which will be discussed later.

### 4.5. Zinc-Mediated Regulation of NFκB Signaling and A20 Expression

In addition to MAPKs, signaling in innate immune cells centers on NFκB. This molecule is involved in regulating a large set of genes, thereby affecting apoptosis, proliferation, cell adhesion, pro-inflammatory responses, tissue remodeling and stress-responses [21]. Important molecules within these signaling pathways include RelA (p65), RelB, c-Rel, p50/p105 (NFκB1) and p52/p100 (NFκB2), which can form various homo- and heterodimers. Inhibitors of NFκB (IκBs) can bind to single molecules and complexes, causing their sequestration in the cytoplasm, hindering the translocation to the nucleus as well as subsequent DNA binding and gene activation. Inhibitors are IκBα, IκBβ, IκBε, IκBγ, Bcl-3, p100 and p105 [36]. The complexity of this signaling process makes its investigation regarding an association to zinc homeostasis a difficult task. Existing studies show conflicting results and a general unilateral statement on the connection is difficult to formulate.

**Figure 2:**
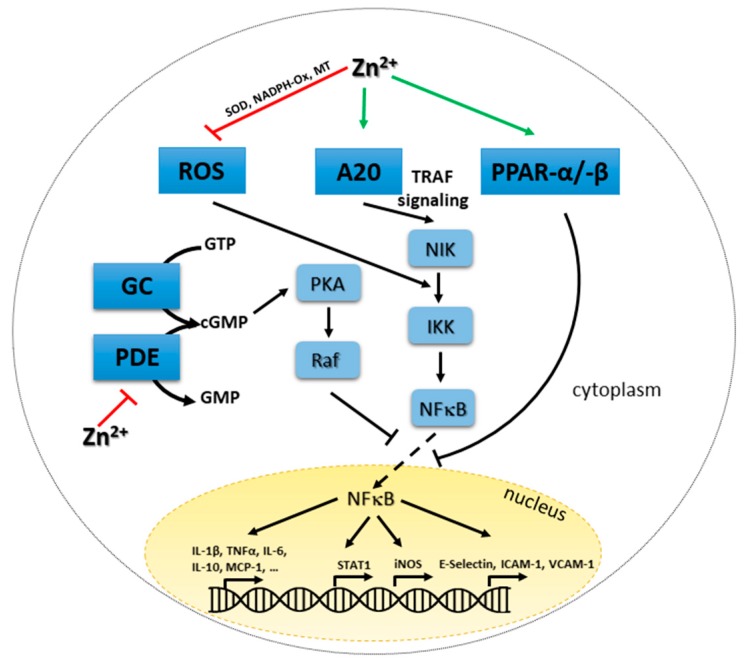
Complex impact of zinc on NFκB-centered signaling as illustration of signaling pathways described in the text. Red T bar: inhibitory function of zinc, green arrow: activating function of zinc, black T bar: inhibition, black arrow activation. Abbreviations: cGMP: cyclic guanine monophosphate; GC: guanine cyclase; GMP: guanine monophosphate; GTP: guanine triphosphate; ICAM: intercellular adhesion molecule; iNOS: inducible nitric oxide synthase; IKK: IκB kinase; IL: interleukin; MCP: monocyte chemoattractant protein; NIK: NFκB-inducing kinase; NFκB: nuclear factor (NF)κB; PDE: phosphodiesterase; PKA: protein kinase A; PPAR: Peroxisome proliferator-activated receptor; ROS: reactive oxygen species; STAT: Signal transducer and activator of transcription; TNF: tumor necrosis factor; VCAM: vascular cell adhesion molecule.

Diverse effects of zinc on NFκB signaling have been observed (see Figure 2) [136].

Chelating zinc using TPEN abrogated LPS-induced, NFκB-mediated signaling [102]. In contrast, zinc has been found to be inhibitory for NFκB signal transduction as well by others [137]. Clear mechanistic insights are not available, but suggestions include the effect of zinc on A20, a principally anti-inflammatory zinc-finger protein, important for tumor necrosis factor receptor (TNFR)- and TLR-induced signaling. A20 can de-ubiquitinate receptor interacting protein (RIP)-1, preventing its binding to I kappa B kinase (IKK)γ, which keeps sequestering NFκB in the cytoplasm. In addition, A20 can remove polyubiquitin chains from TNFR-associated factor (TRAF)6, thereby inhibiting TLR-mediated signaling.

In endothelial and pre-monocytic cells, zinc-dependent induction of A20 mRNA expression and protein production was demonstrated, while information on the ubiquitination activity of the enzyme regarding zinc availability is not clear yet. Zinc signals upregulated mRNA expression of A20 in other immune cells as well [30,69,138]. Inhibition of gene expression by zinc via increased A20 and decreased NFκB activity was found for various molecules, including decreased expression of interleukin (IL)-1β and TNFα in myeloid cells and decreased C reactive protein (CRP) levels, lipid peroxidation and inflammatory cytokines in elderly subjects [53,88,138,139,140]. In addition to A20, peroxisome proliferator-activated receptor (PPAR) alpha is of interest as it alters the binding of NFκB to the DNA. Its zinc-induced expression results in decreased NFκB binding and down regulation of pro-inflammatory cytokine and adhesion molecule expression. [53]. NFκB is also inhibited by PKA via zinc-induced alteration of cGMP levels, as described earlier adding one more explanation for the decrease in NFκB induced gene expression during high zinc conditions and its increase during zinc deficiency [133]. The complex postulated effects of zinc on NFκB centered signaling pathways are summarized in Figure 2.

### 4.6. Zinc and Signaling Proteome

Most of the so far described effects of zinc in signaling depict consequences of immediate changes in zinc homeostasis. Long-term alterations in intracellular zinc are often related to altered expression of zinc transporters and zinc binding proteins. In a recent approach, Aude-Garcia et al. [68] cultured macrophages with zinc oxide nanoparticles or an equivalent dose of zinc acetate for at least 24 h and analyzed the proteome of the cells via 2D gel based analyses. In addition to changes regarding energy, mitochondrial, cytoskeletal and DNA-control metabolism, authors found altered levels of certain signaling molecules. Protein phosphatase 1a and 2a and S/T phosphatase were affected by zinc acetate. Zinc oxide nanoparticles changed the levels of Prostaglandin reductase 2, Phosphatidylinositol transfer protein (PITP)α, Acyl-protein thioesterase 2, Regulator of G-protein signaling 10, MOB kinase activator 1B and Inositol-3-phosphate synthase 1. Protein levels of MyD88 were affected by both the zinc ion and the nanoparticle in a similar manner [68]. If altered proteome is due to changes in gene expression, posttranscriptional mechanisms or if protein stability is reduced remains open. Furthermore, information on whether proteins are functional is missing. However, results suggest that certain signaling pathways are prioritized during high zinc conditions and are important to the cell. MyD88 might be stabilized to keep signaling pathways, important for the pro-inflammatory activity of the cell active during high zinc conditions. The study provides molecules to the list of zinc-regulated targets in signaling, but as protein species-based proteomics have some limitations, results have to be verified carefully [68]. Effects of zinc deficiency should be investigated using a similar approach as well.

### 4.7. Zinc and Transcription Factors

As a note, many transcription factors governing innate immune cell functions are zinc finger proteins, suggesting an additional direct or indirect role of zinc in intracellular signaling. Promyelocytic leukemia zinc-finger (PLZF) is a critical transcription factor for induced natural killer T cell (iNKT cell) development as is PU.1 for myelopoiesis [141,142]. GATA-4, -5, and -6 and krüppel like factor (KLF)-4 and KLF-5 are additional examples of zinc finger transcriptionfactors important during the development of innate immune cells [143,144]. The same is true for adaptive immune cells: zinc finger transcription factors (ZEB1, ZNF292, and ZNF644) were confirmed to play a role in CD4^+^ T cells in Rheumatoid Arthritis. A lot of genes involved in regulating zinc homeostasis reveal metal response elements in their promoters and can be induced by binding of metal-response element binding transcription factor (MTF)-1, which acts as an intracellular zinc sensor, suggesting feedback mechanisms [145]. After some initial analysis in this area of research, this topic has not been followed much recently, but would be a great area for future experiments.

## 5. From Altered Zinc Homeostasis via Signaling to Innate Immune Cell Functions

As described above, the effects of zinc homeostasis on signaling are complex. Knowing the effect of zinc on single signaling molecules does not enable prediction of the functional outcome of zinc deficiency or supplementation. In the upcoming paragraphs, examples will be described for some selected zinc-dependent key functions of innate immune cells. Predicted underlying molecular mechanism for the particular setting will be mentioned as well.

### 5.1. TLR4 Signaling in Monocytes

One of the best-studied examples for the role of zinc in innate immune signaling is TLR4 triggering by LPS. Pathways involved are illustrated in Figure 1 and molecules where zinc is potentially intervening in the signaling pathways are indicated. Long-term zinc deficiency augments pro-inflammatory cytokine production, which is reversible if zinc is reconstituted [48]. In contrast, within two minutes after LPS binding to the receptor complex a significant increase in intracellular zinc can be detected, which is involved in activation of MAPK signaling pathways. Amongst others, zinc inhibits dephosphorylation of MAPK and supports IKKα/β phosphorylation, NFκB translocation to the nucleus and gene activation [102,146]. Wan et al. recently separated zinc-dependent and -independent processes shortly after LPS triggering of TLR4 of murine macrophages. Depletion of zinc inhibited the LPS-induced activation of several kinases including ERK1/2, IKKβ, MKK3/6 and IκB in murine primary macrophages and cell lines. Interestingly, phosphorylation and ubiquitination of IRAK1 was not affected by zinc deficiency, at least not early after stimulation, suggesting a selective effect of zinc on certain signaling pathways, shortly after LPS-stimulation. However, degradation of ubiquitinated IRAK1 required zinc but did not involve proteasome [70]. Zinc chelation caused the accumulation of phosphorylated and ubiquitinated IRAK1. One can speculate, that inactivation of zinc-containing metalloproteases during zinc deficiency, prevents the destruction of IRAK1, which remains to be investigated. A membrane associated complex, which is phosphorylated after LPS stimulation, contains IRAK1 in addition to IKKα and IKKβ [147]. Degradation of IRAK1 is essential to release phosphorylated IKKs into the cytoplasm, which then degrade IκB and p105 activating NFκB and ERK signaling. However, zinc deficiency might affect phosphorylation of ERK and IκB via its effect on PTPs as well, which is not clear yet.

TLR4-induced pathways can be grouped into MyD88-dependent pathways, including MAPKs and NFκB, which induce expression of inflammatory cytokines and MyD88-independent/Toll-interleukin-1 receptor (TIR) domain-containing adaptor-inducing interferon (TRIF)-dependent pathways involving TRIF-related adaptor molecule (TRAM), TRIF and IRF-3 and triggering interferon (IFN)β expression (see Figure 1). The latter has an autocrine function and can bind to surface interferon receptor (IFNR) resulting in activation of the JAK-STAT pathway finally causing expression of CD40, CD80 and CD86, necessary for interaction of myeloid cells with T cells [5,148]. Zinc has been suggested to be involved in IFN signaling in hepatocytes recently, which should be tested for immune cells as well [149]. In addition, NFκB as well as the JAK-STAT pathway induce NO production via inducible nitric oxide synthase (iNOS), which is a potent anti-microbial mediator. While our knowledge on the role of zinc in MyD88-dependent pathways, especially early after receptor triggering, is broad and described in large detail [102], an association of zinc to TRIF pathways and NO synthesis is less explicit. In addition, most data originate from investigations of time points early after receptor triggering, representing investigation of the zinc flux. Pre-existing zinc deficiency augmented LPS-induced NO synthesis in murine macrophages and also increased LPS induced IFNβ expression as well as CD80 and CD86 (cell-line and bone marrow based). Effects were measured a few hours after the original stimulus and where therefore assigned to be regulated by homeostatic zinc signals. Reconstitution of zinc abolished the effect of the chelator. Interestingly, chelation of zinc caused decreased LPS-induced transcription of IL-1β, IL-6, and IL-10 in macrophages compared to zinc adequate controls. This indicates zinc chelation seems to inhibit the MyD88-depending pathways while triggering TRIF-dependent signaling. In line with this, a time-course experiment revealed, that adding the chelator at the time of TRIF activation caused the maximal increase in NO synthesis. This also indicates that TRIF-signaling is independent from the initial zinc signal, induced quickly after LPS binding to TLR4. MyD88-dependent pathways induce fast translocation of NFκB to the nucleus which was reduced, when cells were pre-incubated with TPEN. However, TRIF-dependent delayed NFκB activation was not affected by TPEN, underlining this hypothesis [5].

Regarding effects of persisting changes in intracellular zinc levels, the de-ubiquitination of TRAF6 by A20 is probably involved in turning off TLR4-induced signaling and pro-inflammatory activity of macrophages. Ubiquinated TRAF6 activates TAK1 a kinase phosphorylating IKKs and MKKs, resulting in gene expression which is thereby blocked [138,150,151]. Thus, balanced zinc homeostasis is important to prevent unwanted signaling disturbances. In macrophages, A20 is induced by pro-inflammatory cytokines, possibly as a feedback mechanism to terminate their expression [152]. Increased cGMPs levels due to high intracellular zinc could explain elevated phosphorylation of Raf-1, mediating its inactivation as well as inactivation of NFκB as can be seen in Figure 2. Investigation of TLR7 and TLR3-induced signaling showed dependents of TRIF/IRF/IFNβ signaling as well, while NO production was not affected by zinc chelation after TLR3 activation.

Altogether, TLR4 signaling in macrophages is a great example of the combination of a short-lived zinc peak and modulation of basal intracellular zinc levels for longer periods to balance MyD88- and TRIF-dependent pathways, acting as a fine-tuning signal after TLR4 triggering. Using ligands for other TLRs including Pam3CSK4 (TLR1/2), Listeria monocytogenes (TLR2), flagellin (TLR5), FSL-1 (TLR6/2), ssRNA40 (TLR7) and ODN1826 (TLR9) all increased intracellular zinc in murine macrophages and primary human monocytes [5,102]. If this has similar consequences on signaling pathways remains to be investigated. Simplification of zinc’s effect such as stating that zinc generally supports all TLR-induced signaling pathways, is difficult, as the whole environmental set up, including the current zinc supply, activation and maturity state of the cell, chronic inflammation, time after stimulation and more, can affect consequences of altered zinc levels.

### 5.2. Zinc Homeostasis, Hematopoiesis and STAT3

Long-term changes in zinc homeostasis usually involve changes in zinc transporter expression. In dendritic cells, stimulation with LPS changes the expression of Zip6 and Zip10 as well as of ZnT-1, -4 and -6 via pathways involving TRIF. An association of zinc with regulation of TRIF signaling has just been described above, explaining this observation. This causes a long-term reduction in intracellular zinc which is essential for cellular maturation [58]. This is just one of the examples explaining the often-observed effect of zinc on hematopoiesis. Monopoiesis has been shown to be paralleled by a decrease in intracellular zinc and similar data have just been generated for granulopoiesis [38,153].

IL-6 is one of the most potent inflammatory cytokines, activating immune cells via binding to its gp130 receptor found within the plasma membrane. Amongst others, IL-6 induces the expression of acute phase proteins (APP), which subsequently interact with TLRs and induce the NLRP3 inflammasome. Receptor ligation activates JAK-STAT3 and MAPK pathways. Recent years revealed that especially the phosphorylation of STAT3 is broadly affected by zinc homeostasis. Zinc deficiency increased IL-6-induced activation of JAK-STAT3 signaling pathways; reconstitution with zinc normalized the effect while zinc supplementation was shown to inhibit STAT3 phosphorylation induced by IL-6 and IL-1 [111]. Phosphorylation of STAT3 is known to be regulated by SHP1/2, whose activity is increased by zinc [154]. If this is the only mechanism how zinc is involved in JAK-STAT signal transduction remains to be further investigated. STAT3 seems to be an important player in regular myelopoiesis as well as in demand adapted emergency myelopoiesis, during inflammatory diseases such as sepsis [155]. The connection between disease-related serum hypozincemia, STAT3 activity and grade of differentiation needs to be explored.

In murine hematopoietic stem cells, zinc chloride promotes transient STAT3 phosphorylation (Tyr705), resulting in increased expression of pluripotency genes and inhibition of differentiation genes [112]. Expression of STAT3 was not altered. Thus, zinc plays a critical role in maintaining pluripotency and self-renewal of stem cells and can even replace leukemia inhibitory factor (LIF), which is normally essential for this process. Zinc disrupted differentiation induced by retinoic acid, at least for a certain period of time. Treatment of cells with zinc chloride and LIF did not augment pluripotency, suggesting that both factors regulate the same pathway, which is centered on STAT3. The observation that inhibition of STAT3 abrogates the effect of zinc chloride on the cells underlines the importance of STAT3 as a central molecule in hematopoiesis. Along with this, B cell proliferation, was increased during zinc deficiency [77]. Again, STAT3 phosphorylation was shown to be responsible here.

Neuronal development is also strongly affected by zinc via STAT3-centered signaling. Results from this very recent study add alteration in the redox-state as underlying mechanism for the effect of zinc on STAT3 phosphorylation. In this study, moderate zinc deficiency (1.5 µM) caused oxidative stress within 24 h, resulting in decreased tyrosine phosphorylation and retention of STAT3 in the cytosol. In addition to tyrosine (Y705), serine (S727) phosphorylation was analyzed. While only tyrosine phosphorylation was decreased during zinc deficiency when whole cell lysates were tested, in nuclear extracts phosphorylation of both residues was decreased [156]. If this is a cell-type specific effect or if it can be extrapolated to innate immune cells remains to be tested, but as redox and zinc metabolism are highly intertwined in innate immune cells, as described earlier, a similar mechanism is highly likely.

Thus far, an association of zinc homeostasis with the activity of STAT molecules other than STAT3 is barely investigated. The effect of zinc on phosphorylation of STAT1 and STAT6 was suggested to be relevant for lineage determination of macrophages. Zinc supplementation decreased STAT6 phosphorylation reducing the amount of M2 macrophages, while zinc deficiency increased phosphorylation of STAT2, thereby supporting M1 development [78]. While studies in T cells did not show an effect of zinc on STAT5 activity, recent results from myeloid cells suggest that zinc supplementation inhibits STAT5 phosphorylation [157]. This indicates a general mechanism of zinc affected STAT activity in cell differentiation, which should be investigates in more detail.

### 5.3. Zinc Alters Killing Activity of Natural Killer Cells

Although natural killer cells belong to the lymphoid lineage, they are counted as innate immune cells and kill infected or transformed cells. Regular human cells carry major histocompatibility complex (MHC)-I on their surface, which rescues them from being killed by binding to p58 killer cell inhibitory receptor (KIR) on NK cells. KIR can bind zinc which alters recognition of MHC-I and thereby the induction of subsequent signaling pathways. A zinc-dependent multimerization of KIR was suggested to be essential for formation of the clusters of KIR and human leukocyte antigen (HLA) molecules, also called “natural killer (NK) cell immune synapse” [84,158]. Effects of zinc deficiency on KIR multimerization have not been investigated, yet, but might explain why the lytic activity of NK cells is decreased when zinc supply is limited [159]. In general, existing studies for NK cells are limited to experiments, where zinc has been extracellularly added, whereas effects of altered intracellular zinc homeostasis can only be assumed, as measurements of intracellular zinc levels after stimulation are not available. Only some very recent data are available that showed a zinc flux shortly after IL-1 stimulation [85]. However, NK cells carry a variety of cell surface receptors, essential for fine tuning of their activities, which contain tyrosine phosphorylation sides such as immunoreceptor tyrosine-based activation motif (ITAM) or immunoreceptor tyrosine-based inhibitory motif (ITIM). As described earlier, the ability of zinc to inhibit PTPs is well accepted, so a role of intracellular zinc homeostasis in regulation of signaling pathways in NK cells is highly likely, but remains to be analyzed.

### 5.4. Mast Cell Degranulation Depends on Zinc Levels

Knowledge of a role of zinc in signaling in mast cells (MCs) is scarce. Cytoplasm and granules seem to represent two distinct zinc pools in this cell type. High amounts of zinc are stored in the granules, lost during immunglobulin (Ig)E-induced degranulation, and are quickly replenished thereafter. This zinc is probably not involved in signaling [160]. Zinc was however important for the process of degranulation, mediating granule translocation to the plasma membrane Zinc chelation inhibited degranulation. A transient increase in cytoplasmic zinc has been reported after cell activation [161]. This zinc signal affected translocation of NFκB to the nucleus and phosphorylation of ERK1/2, JNK1/2 and the translocation of PKC to the plasma membrane, as reported for other cells and stimuli [55,161]. Abrogation of the zinc wave via incubation with the zinc chelator TPEN abrogated activation of signaling pathways and blocked expression of IL-1β and TNFα. Zinc supplementation was able to prolong activation of the signaling pathways. Different probes were used to measure zinc here, enabling staining of the different zinc pools. It is well known, that low molecular weight probes highly differ in their exact intracellular location, which might explain the discrepancies [162].

## 6. The Adaptive Immunity

The adaptive immune system is built up of the humoral immune defense, represented by B lymphocytes (B cells), and the cellular immune defense, represented by T lymphocytes (T cells). Both cell types evolve off precursors and are educated to recognize their specific antigen in the thymus (T cells) or bone marrow (B cells), respectively. According to antigen contact, naïve lymphocytes differentiate into effector cells or memory cells, facilitating a stronger reaction to a known antigen as a secondary response due to immunological memory. The immune system is a highly active and proliferating organ influenced by a multitude of external factors such as nutrition. In this regard, the immune response is highly dependent on zinc, since zinc deficiency is accompanied with malfunctions and disorders, such as allergies, autoimmune diseases and cancer. In the past decade, studies on animal models have provided considerable knowledge about the underlying molecular mechanisms of the zinc-related modulation of the immune system, including new insights into how nutritional deficiency can alter immune cell homeostasis and function of the adaptive immunity. Zinc-related immunological changes in the adaptive immune response and altered signaling cascades are discussed in the following sections.

### 6.1. Zinc Homeostasis in Development of T and B Cells

For a long time, zinc is known to be essential especially for proper T cell and B cell development [163,164]. However, T cells are in particular much more sensitive to altered zinc signals resulting in altered differentiation or function, since zinc directly influence the biological activity of the thymic hormone thymulin by acting as essential co-factor. Thymulin, is produced by the thymus and released by thymic epithelial cells [165]. By binding to high-affinity receptors on T cells, it induces markers of differentiation in immature T cells, and promotes T cell function, including allogenic cytotoxicity, suppressor functions, and IL-2 production [166]. It is therefore not surprising that during zinc deficiency the recruitment of naïve Th cells and the percentage of cytotoxic T lymphocytes (CTL) precursors is diminished respectively [167]. The loss of developing T cells (pre-T cells) is ascribed to accelerated apoptosis consistent with the finding that pre-T cells express the lowest amount of the anti-apoptotic proteins B cell lymphoma (Bcl)-2 and Bcl-xL thereby increasing the vulnerability towards cell death. In general, zinc deficiency is described to affect for instance the hypothalamus-adrenal-pituitary axis, resulting in increased circulating glucocorticoids, being powerful apoptogens for immature lymphoid cells [168]. Additionally, caspase activity is known to be regulated in a zinc-dependent manner and zinc deficiency directly induce caspase 3-dependent cleavage of the cell cycle regulator p21 leading to an immediate induction of cyclin-dependent kinase (CDK)2 activity which may result in premature entry of the cells into S-phase and apoptotic cell death [169,170]. Since the thymus comprises of about 80% pre-T cells, its strong susceptibility towards zinc deficiency account for a main cause of disturbed T cell-mediated immune responses. Interestingly, in vitro and in vivo supplementation of serum zinc restored thymulin activity observed in moderately zinc-deficient mice and men, emphasizing a direct effect of serum zinc on thymulin activity [165,171].

Besides intrathymic functions on thymocytes and immature T cells, mature T cells in the periphery are also affected by thymulin. Herein, thymulin modulates cytokine release by PBMC, and proliferation of cytotoxic CD8-expressing CTL in combination with IL-2 responsible for T cell activation and proliferation [172]. Hence, it is obvious that zinc status has impact on immature as well as mature T cells through the direct activation of thymulin. As a consequence of zinc deficiency, T cell proliferation is decreased after mitogen stimulation, whereas zinc supplementation is able to reverse zinc deficiency-induced changes in the thymus and on peripheral cells [173].

**Figure 3:**
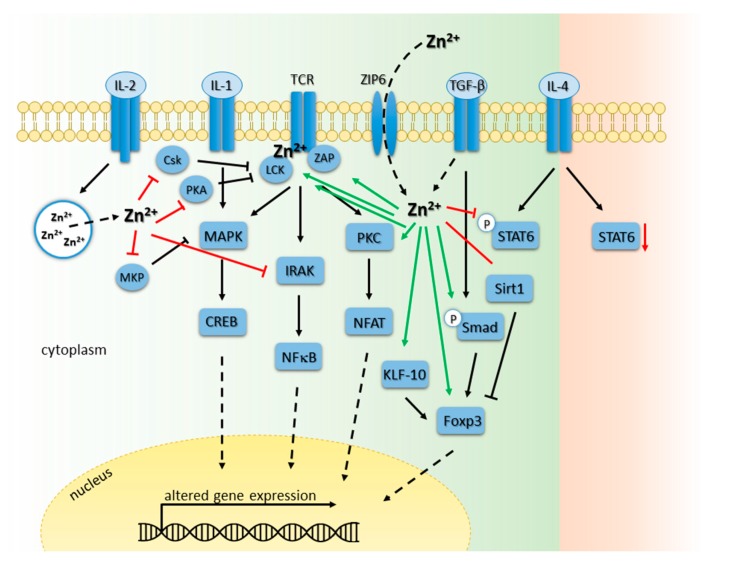
Influence of zinc signals on T cell signaling pathways. This figure presents an overview of T cell receptor (TCR)-, Interleukin-1 receptor (IL-1R)-, IL-2R-, IL-4R-, Transforming growth factor β1 receptor (TGF-β1R)-signaling in T cells, as well as zinc flux via zinc transporter ZIP6. Signaling pathways are described in detail in the text. Black arrow: activation, green arrow: activating function of zinc, red T bar: inhibiting function of zinc, black dotted arrow, translocation of the molecule to nucleus. Abbreviations: CREB: cyclic adenosine monophosphate response element-binding protein; Csk: c-src tyrosine kinase; Foxp3: forkhead-box-protein P3; IL: Interleukin; IRAK: interleukin-1 receptor-associated kinase; KLF-10: krüppel-like factor-10; Lck: lymphocyte-specific protein tyrosine kinase; MAPK: mitogen-activated protein kinase; MKP: MAP-kinase phosphatase; NFAT: nuclear factor of activated T cells; NFκB: nuclear factor kappa B; PKA: protein kinase A; PKC: protein kinase C; SIRT1: Sirtuin1; STAT: signal transducer and activator of transcription; TCR: T cell receptor; TGF-β1: transforming growth factor β1. ZAP: zeta-chain (TCR)-associated protein kinase.

In general, both mature T cell types: CD4-expressing Th cells as well as CD8-expressing CTL, are affected by zinc deficiency. On the one hand, the amount of CD8/CD73 co-expressing T cells is decreased during zinc deficiency. Those are predominantly precursors of cytotoxic T cells, needing CD73 expression for antigen recognition and proliferation as well as cytolytic process generation [167]. On the other hand, polarization of mature Th cells is disturbed. Polarization into Th1 cells is impaired and therefore changes in the Th1/Th2 ratio towards Th2 cells is observed leading to unbalanced cell-mediated immune responses [174]. Herein, Th1 cell products as IFNγ and IL-2 are decreased, whereas Th2 cell products like IL-4, IL-6, and IL-10 remain unaffected. Therefore, the incidence of infections and Th2-driven allergies is increased by zinc deficiency [23,175]. Furthermore, the functional impairment of T cell-mediated responses during zinc-deficiency is described to favor the development of autoimmune diseases [20,176]. In this regard, patients suffering from multiple sclerosis (MS) show lower plasma zinc level and lower zinc concentrations in chronic MS lesions [14,177,178]. Moreover, adequate zinc status is essential for transplant rejections since zinc supplementation was shown to beneficially influence allogeneic cardiac transplantation and intraportal islet transplantation [87,179]. Here, zinc as a pro-antioxidant is involved in the inhibition of apoptotic enzymes such as caspases and acts tolerogenic [79,87,180]. Another mechanism to lower transplant rejections by zinc administration in physiological doses is the capacity of zinc to induce and stabilize a specific subpopulation of Th cells, namely regulatory T cells (Treg) in vitro and in vivo [79,80,81]. This is of great importance, since the discovery of Treg offers a new paradigm for transplantation medicine since intra-graft Treg frequency seems to correlate with clinical graft acceptance, survival, and function [181,182]. Moreover, pro-inflammatory graft-related immune reactions are tried to impede that might be accomplished by Treg induction. To date, Treg gain more and more importance in combating diseases in clinical approaches and a multitude of studies elicited transforming growth factor (TGF)-β1 to be essential in conversion of Th cells into Treg in vitro [183,184,185]. In this connection, TGF-β1 seem to be important in establishing immunological tolerance by induction of Treg cells in mice [183,186,187] and humans [185,188] by triggering the TGF-β1-dependent Smad signaling pathway. Herein, studies suggest an essential role for Smad 2/3 in Forkhead-Box-Protein (FoxP)3 induction and cytokine suppression [189,190]. Interestingly, TGF-β-induced Smad signaling was shown to be intensified by zinc administration contributing to higher Treg cell induction (see Figure 3). Because Smad-binding elements were found in the conserved non-coding DNA sequence (CNS) 1 region of the FoxP3 promoter [189], and zinc promoted FoxP3 stability by preventing proteasomal degradation caused by the histone deacetylase Sirt1 [79], the synergistic effect of the combined treatment could result from both triggered mechanism.

Another mechanism controlling Treg function probably is CK2-mediated activation of zinc transporter Zip7 and thereby altering cellular zinc homeostasis. Zip7 is considered as gatekeeper of cytosolic zinc release from the ER [191], highlighting the involvement of zinc in rapid signaling. Protein kinase CK2 is a ubiquitously expressed tetrameric threonine/serine kinase composed of two catalytic α-subunits and β-subunits respectively [192]. CK2 particularly is involved in regulation of cell survival and proliferation, apoptosis and mitosis [193], by shuttling between the cytosol and nuclei of cells in order to support apoptosis or mitosis, respectively. Zip7 phosphorylation by CK2 results in Zip7-mediated zinc release from the ER and the subsequent activation of multiple downstream pathways that enhance cell proliferation and migration. In the context of Treg function, genetic ablation of the β-subunit of CK2 is addressed to insufficient potential to suppress allergic immune responses in the lungs mediated by Th2 cells in vivo [194]. Therefore, inappropriate CK2 function impairs cell-specific immunological tolerance that might be due, amongst others, to changed zinc homeostasis.

In contrast to that, the ZIP8-zinc axis might have a negative impact on immune regulation. ZIP8 is known to be highly expressed in human T cells on the lysosomal membrane and is even more prominent when T cells are activated by TCR triggering [125]. Hence, after T cell activation free intralysosomal zinc is released into the cytoplasm increasing the zinc available, which leads to altered T cell function by modulation of distinct signaling pathways. In this connection, overexpression of ZIP8 was shown to trigger the pro-inflammatory immune responses by enhancing the IFN-γ production due to zinc-mediated reduction of CN phosphatase activity and prolonged phosphorylation of the transcription factor cyclic adenosine monophosphate response element-binding protein (CREB) [125]. Hence, the TCR-induced cytokine production is strengthened. In line with that, the NFκB activity is triggered by downregulation of the IκB kinase activity in pro-inflammatory responses observed in ZIP8 hypomorphic mice [137]. Hence, an overreacting immune system can lead to hyperinflammation, autoimmune diseases or sepsis.

Nevertheless, one has to keep in mind that T cell function is sensitively regulated by zinc concentration leading to cellular activation or deactivation respectively [31]. Additionally, results observed in different species and different cellular models needs to be compared with wariness.

In general, Treg development and survival is dependent on a high number of key factors, such as the transcription factor Foxp3 and additional signals, including IL-2, TGF-β, and co-stimulatory molecules like CD28. Moreover, transcription factors as IRF-1 and KLF-10 are described to influence Treg stability and function. One study elicited IRF-1 deficiency to result in a selective and marked increase in highly differentiated and activated FoxP3 expressing Treg cells in vivo [195]. IRF-1 plays a direct role in the generation and expansion of Treg cells by specifically repressing FoxP3 activity that can be dampened by zinc supplementation supporting the pro-tolerogenic immune reaction [196]. In contrast to IRF-1, KLF-10 seem to be indispensable for appropriate Treg function, because animals carrying a disruption in KLF-10 no longer show FoxP3 activation [197]. KLF-10-deficient Treg cells display impaired cell differentiation, altered cytokine profiles with enhanced Th1, Th2, and Th17 cytokine expression. Furthermore, a reduced capacity for suppression by wild-type co-cultured T effector cells, as well as accelerated atherosclerosis in immunodeficient atherosclerotic mice was exhibited [197].

During the last decade, the pro-inflammatory Th9 cell subpopulation is reported to be involved allergic asthma and autoimmune diseases like MS respectively [198,199]. Interestingly, comparable to the zinc-mediated reduction of pro-inflammatory Th17 cells in allogeneic immunoreactions [81,200], zinc supplementation in physiological doses facilitates a similar reduction of pro-inflammatory Th9 cells [82], subsequently leading to dampened allogeneic immunoreaction. Hence, an overreacting immune response can be beneficially influenced by zinc administration seeming to be promising to improve the life of patients suffering autoimmune or allergic diseases.

To date, zinc-related targets for the above-mentioned effects are still not fully known. However, some zinc-related targets for the above mentioned effects were found, comprising receptor proteins, kinases, phosphatases, caspases, and transcription factors. Those can be activated or inactivated by zinc administration or zinc deficiency depending on type of zinc signal and zinc level [4,30]. The impact of zinc signals on the adaptive immune system will be discussed in detail in the following paragraphs.

### 6.2. Zinc Signals in T Cell Receptor-Triggered Signaling Cascades

When T cell activation via T cell receptor (TCR) stimulation is examined into detail, already the TCR activating complex is influenced by zinc signals (see Figure 3). One has to keep in mind that TCR exhibit no intrinsic kinase activity and depend on Src-family tyrosine kinases for signal transduction, like lymphocyte protein tyrosine kinase (Lck). Lck is one of the first kinases activated, essential for T cell activation mediated either by proximal or distal Lck promoter activity, and is essential for phosphorylation of the 10 ITAM motifs of the T cell antigen receptor-signaling complex augmenting phosphorylation of the kinase ZAP70 [201,202]. Activation of ZAP70 phosphorylates downstream targets eventually inducing MAPK signaling pathway contributing to T cell activation. Zinc-mediated T cell activation is facilitated by either a zinc flux inducing direct or indirect Lck activation or SHP-1 deactivation, respectively, or homeostatic zinc signals altering gene expression, as for instance of distinct zinc transporter.

Lck activation occurs by zinc flux-dependent Lck homodimerization by stabilization the dimer interface of the SH3 domains [67]. Herein, the activation is highly complex by involving distinct tyrosine residues either in the so-called activation loop or at the C-terminal negative regulatory site [154,201]. On the other hand, Lck activation is facilitated by stabilization the interface site of Lck and the membrane proteins CD4 and CD8 respectively [63,64]. By forming a so-called “zinc clasp structure”, zinc bridges two cysteine residues of each protein facilitating a stable interaction of Lck and CD4/CD8 [203]. Since the surface molecules CD8 and CD4 bind to MHC class I or II respectively, all components are recruited in close proximity to the TCR signaling complex, leading to T cell activation. On the other hand, Lck activity is influenced in an indirect manner due to reduced recruitment of the enzyme SHP-1. Since Lck homodimerization and phosphorylation, as well as TCR complex arrangement and signaling, is highly dependent on numerous PTP [204,205], all can be considered as potential targets for zinc-mediated regulation. Consequently, it cannot be precisely predicted whether a PTP regulation result in preferential dephosphorylation of an activating or inactivating tyrosine by zinc in vivo eventually facilitation signaling activation or inactivation.

T cell activation can furthermore be influenced by altered intracellular zinc signals and zinc concentrations mediated by specific zinc transporter, as for instance Zip6. Initiation of TCR signaling is initiated by specific interaction with an antigen-loaded MHC molecule on the surface of a neighboring antigen-presenting cell (APC) by formation of a functional immunologic synapse. This results in an immediately influx of zinc from the extracellular environment through the transporter Zip6 after T cell stimulation [66]. Hence, intracellular free zinc levels are altered influencing signaling pathways and subsequently T cell activation, maturation and differentiation.

In response to TCR stimulation, specific transcription factors and signaling pathways are triggered that are often zinc-regulated (see Figure 3). Nuclear factor of activated T cells (NFAT) mediates the expression of plenty of genes, such as IL-2. In resting T cells, NFAT proteins are constitutively phosphorylated remaining inactive and are located in the cytoplasm. In response to TCR/CD28-mediated calcium signaling, NFAT is dephosphorylated by calcineurin (CN), a calcium/calmodulin-dependent serine/threonine phosphatase, and eventually translocates into the nucleus [206]. Zinc and iron are essential cofactors for the catalytic domain of CN, containing a Zn^2+^-Fe^2+^ binuclear center. However, CN inhibition is only reported for zinc. Regarding this, physiologic zinc concentrations, ranging from 10 to 10 μM are described to exhibit a CN inhibition-capacity in vitro [116,207]. Zinc-dependent regulation of T cell activation, proliferation and differentiation is much more complex than modifying transcription factor activity as NFAT, since CN activity itself is regulated in a complex zinc-dependent manner. Herein, CN remains in its inactive phosphorylated form, due to phosphatidyl-inositol-3-kinase (PI3K) [206]. PI3K is a lipid kinase and generates phosphatidylinositol-3,4,5-trisphosphate (PI(3,4,5)P3).

PI(3,4,5)P3 acts as second messenger essential for the translocation of Akt to the plasma membrane where it is phosphorylated and activated by phosphoinositide-dependent kinase (PDK)1 and PDK2. Activation of Akt plays a pivotal role in fundamental cellular functions such as cell proliferation and survival by phosphorylating a variety of substrates [208]. In PI3K pathway, zinc signals are well reported to influence the signaling cascade in various cell types [209,210,211,212]. One possible explanation for this mechanism is an increased enzyme degradation of phosphatase and tensin homolog deleted on chromosome 10 (PTEN) [213,214]. PTEN in general functions as a dephosphorylase of PI(3,4,5)P3, a product of PI3K mediating the activation of PDK-1/Akt. More recently, a study examining the IL-2-induced PI3K/Akt signaling pathway uncovered an inhibition of PTEN. Here, homeostatic zinc signals seem to be necessary for the regulation the PI3K/Akt pathway, since the IL-2-induced Akt phosphorylation is diminished during zinc deficiency. Homeostatic zinc signals are known to upregulate phosphorylation of Akt at Ser473 and inhibit PTEN at subnanomolar concentrations (IC_50_~0.59 nM). This inhibition seems to be mediated by zinc binding to cysteine thiol at position 124 (Cys124) essential for the catalytic activity of PTEN [76]. Thus, a modulation of this pathway occurs upstream of Akt, but down-stream of Jak1, because STAT-5 signaling is not influenced by zinc signals [65]. These results suggest a comparable activation of PI3K via zinc in T cells. Zinc-mediated inhibition of CN results in NFAT inactivation and reduction of TCR-mediated transcription, whereas activation of PI3K signaling act agonistic.

Zinc signals as mediator of T cell signal transduction is known since the first reports of a potential role of this ion in signaling were described. Its interaction with PKC is identified as biochemical basis of these observations [215]. The PKC family members are serine/threonine kinases comprising of several isoforms are known to play crucial roles in intracellular signal transduction elicited by various extracellular stimuli, like growth factors, hormones, and neurotransmitters. PKC members can be subdivided into: (1) classical PKCs, activated by cofactors like Ca^2+^ and diacylglycerol (DAC); (2) novel PKC that bind DAC but no Ca^2+^; and (3) atypical PKCs that interact with neither Ca^2+^ nor DAC. Several isoforms are well described to be important in T cell function. Following T cell activation via TCR/CD28 triggering, PKCθ is involved in the activation of several transcription factors. Additionally, PKCα is involved in T cell proliferation and IL-2 production. Furthermore, several PKC isoforms are involved in survival of B cells, pre-B cell development, and induction of tolerance toward self-antigens [216]. PKC is described as a zinc metallo-enzyme. Atomic absorption measurements on the intact enzyme indicated that four zinc atoms (4.2 ± 0.5) are bound per PKCα molecule. Similar stoichiometric ratios were determined for PKCβII and PKCγ [217]. PKC comprise of four commonly conserved domains (C1-C4). Herein, DAC binds to C1 domain in the N-terminal regulatory part of PKCα1, which contains two homologous regions containing six cysteine (Cys) and two histidine (His) residues, forming a total of four Cys3His zinc binding motifs [218]. No information about a differential effect of zinc on the different isoforms is available, and varying forms of C1 domains are present in conventional, novel, and atypical PKCs, pointing to probable zinc binding to all known PKC isoforms [216].

Late zinc signals are mentioned to affect multiple steps during PKC activation, as for instance augmented PKC kinase activity, increased affinity to phorbol esters, and enhanced binding to the cytoskeleton and plasma membrane [219]. Inhibition of the above mentioned events can be seen during zinc deficiency in vitro, facilitated by membrane-permeable zinc chelators, as TPEN. Interestingly, PKC itself can be a source for zinc release, thus interaction between PKC and zinc is not limited to an effect of zinc on the PKC activation. PKC activation by lipid second messengers or thiol oxidation lead to measurable zinc release from the regulatory domain [220,221]. In addition, PKC regulates the intracellular free zinc concentration and distribution. In T cells, phorbol ester treatment lead to a zinc flux resulting in redistribution of zinc from the nucleus and mitochondria to the cytosol and microsomes [222].

Besides activating signaling cascades, also inhibition of TCR signaling and subsequent inhibition of overall T cell activation is facilitated by phosphorylation status of different amino acids. One of those is phosphorylation of the inactivating tyrosine 505 of Lck via c-src tyrosine kinase COOH-terminal Srk kinase (Csk) [223]. The zinc flux interferes with this event in different ways: (1) zinc signals lead to an inhibition of Csk [75]; and (2) activation of Csk in T cells can be observed via phosphorylation by PKA [224]. Although zinc has no direct impact on PKA activity itself, it inhibit AC transcription by a homeostatic zinc signal thereby inhibiting the formation of the PKA activator cAMP. Furthermore, cAMP is influenced by zinc via inhibition of PDE that can block degradation of cyclic nucleotides, leading to activation of PKA [60]. An inhibition of Csk and AC promote TCR signaling, while PDE inhibition antagonizes it. However, the outcome on TCR signaling resulting from the modulation of this pathway by zinc in vivo remains to be elucidated yet.

### 6.3. Zinc Signals in Interleukin Receptor Signaling Pathways in T Cells

Besides PI3K and PTEN modulation downstream of TCR activation, zinc signals also affects signals originating from the IL-1 receptor (IL-1R). High zinc concentrations of about 100 µM inhibit IL-1β-stimulated IFNγ production in primary human T cells and IL-1 dependent proliferation of murine T cells. Zinc incubation leads to a reduced activity of IRAK that is a central kinase in the signaling pathways downstream of the IL-1 receptor. IRAK comprise of four different genes existing in the human genome (IRAK1, IRAK2, IRAK3, and IRAK4), and studies with transgenic mice, have revealed distinct, non-redundant biological roles [225]. Interestingly, IRAK4 is reported to be essential for the TLR signaling in innate immunity as well as for TCR-mediated signaling in adaptive immunity leading to the activation of NFκB [226], however this is still matter of debate [227]. Since zinc signals show modulating capacity on TCR as well as on IL-1-dependent signaling via IRAK inhibition, two central signaling pathways are uncovered in negative regulation of T cell activation. Nevertheless, one has to keep in mind that IRAK function and zinc-mediated regulation is not limited to T cells. High similarity between TLR4 and IL-1R signal transduction suggests IRAK inhibition for another mechanism by which zinc negatively influences TLR4 signaling in innate immunity as discussed before.

Moreover, triggering of interleukin receptor (IL-2R) also results in altered intracellular zinc signals. Following IL-2R stimulation, zinc signal is releases of from lysosomal compartment called zincosomes within several minutes after T cell stimulation [65]. Thus, an intracellular zinc flux is triggered influencing PTP, as for instance dual-specificity phosphatases (DUSP) or Protein phosphatase (PP)2A activity in a direct manner. Subsequently, ultimately signal transduction and protein expression, such as diminished dephosphorylation of MAPK MEK and ERK, or elevated transcription of zinc transporter such as Zip6, can be modulated by zinc.

Not only is kinase activity regulated by zinc signals, but cytokine production is also influenced. During zinc deficiency zinc administration leads to a fast rise in intracellular zinc levels, i.e., zinc flux, due to an increased expression of cell membrane-located zinc transporters Zip10 and Zip12 because of the former zinc deficiency. Due to altered intracellular zinc homeostasis, activity of signaling molecules as MAPK p38, and NFκB p65 are affected consequently leading to highly increased IL-2 mRNA expression and IL-2 cytokine production [72].

In general, a forecast of zinc-related effects on T cell function is impossible due to the high number of signaling pathways modulated by zinc signals. Several studies concerning zinc status and T cell function in vivo, stated an increase of the delayed type hypersensitivity reaction upon correction of zinc deficiency [228]. By contrast, zinc administration diminishes the allogeneic reaction in the mixed lymphocyte culture (MLC) [229] and stabilizes regulatory T cell function in vitro and in vivo [79,230] indicating that zinc may have multiple, opposing functions, depending on its concentration and interaction with multiple other environmental factors.

### 6.4. Zinc Signals in B Cell Maturation, Survival and Function

B cells represent the specific humoral immunity and differentiate to antibody-producing plasma cells after stimulation. Nearly all of the zinc regulated signaling pathways discussed above in T cells are also important in B cells maturation and differentiation, such as tyrosine phosphorylation, PKC, MAPK signaling, and activation of the transcription factors NFAT and NFκB. In contrast to other cell types discussed before, mature B cell proliferation and function is not as dependent on the organisms’ zinc status. Hence, the impact of zinc deficiency on B cell maturation and function and its subsequent impact on the immune system is not as profound as it is in the context of T cells [231]. Zinc signals itself seem to have no impact on B cell activity in a direct manner. However, zinc deficiency affect B cell lymphopoiesis in different ways. Following acute zinc deficiency, the B cell compartment is largely reduced (about 50% to 70%) and the composition of surviving B cells within the B cell compartment markedly changed [232]. In contrast, chronic zinc deficiency is reported to merely influence T cell development and B cell development remains sustained [164].

During cellular development, lymphocytes are sensitive for apoptosis signals due to positive and negative selection mechanisms in the primary lymphoid organs. Mature cells are apoptosis resistant and getting inactivated in the case of autoreactivity known as anergy. A strict selection guarantees adequate cellular function, thus avoiding cellular autoreactivity by eliminating the majority of newly formed cells by apoptosis. Zinc deficiency increases apoptosis within the B cell population and leads to cell depletion [11,233]. Thus, there are fewer naïve B cells during zinc deficiency that can react to neoantigens. Contrarily to zinc deficiency, low zinc levels have no influence on the cell cycle status of precursor B cells and only modest influence on cycling pro-B cells. Taking into account that T cell numbers are reduced during zinc deficiency as well, and that recognition of most antigens is T cell dependent, it is probable that the body is unable to respond with antibody production in response to neoantigens. This assumption is consistent with findings showing a disturbed antibody production by B cells during zinc depletion [234]. This disturbed feedback mechanism might also be an explanation for zinc deficient patients, like elderly and hemodialysis patients, showing a reduced response to vaccination [235,236,237]. Another possible mechanism impairing adequate immune responses are epigenetic changes of DNA. The epigenome is highly influenced by environmental changes, but also by nutrition [238]. Since histone modifying enzymes as histonedeacetylases are known to be regulated in a zinc-dependent manner [79], the organism’s zinc status is important for adequate epigenetic modification of DNA.

Studies uncovered B cell activity and antibody production to be impaired during zinc deficiency respectively leading to higher risk of parasitic infection [239]. On a molecular mechanism this might be due to diminished STAT6 phosphorylation during zinc deficiency. Hence, zinc signals are important for proper IL-4 induced STAT6 phosphorylation [77]. Since IL-4 promotes activation of early B cells and immunoglobulin class switch towards IgE, and thereby the further antibody specification, this mechanism explains the higher risk of parasitic infection during zinc deficiency. Furthermore, zinc signals are important for IL-6 induced STAT3 phosphorylation shown to be increased due to zinc deficiency. IL-6 mediates the activation and final differentiation of B cells into plasma cells and IL-6 overproduction is associated with autoantibody production. Diseases such as rheumatoid arthritis and plasma cell neoplasia go along with reduced serum zinc levels, indicating potential co-effects of IL-6 overproduction and enhanced susceptibility of B cells due to zinc deficiency. Those observations indicate that a strict regulation and proper zinc homeostasis is necessary to keep the immune system balanced [77].

Studies reveal that antibody production as consequence to T cell-dependent antigen recognition is more sensitive to zinc deficiency than antibody production in response to T cell-independent antigens [240]. Zinc deficient mice show reduced antibody recall responses to antigens they have been immunized with before. This effect was observed in T cell-independent as well as in T cell-dependent systems. Thus, immunologic memory also seems to be influenced by zinc [241]. However, mature B cells still are more resistant to zinc deficiency-induced apoptosis due to high Bcl2 level. Thus, B cell memory is less affected than the primary response [233]. Homeostatic zinc signals influence several regulatory proteins, such as those from the Bcl/Bax family [11] and furthermore several aspects of apoptotic signal transduction. In this connection, the calcium-dependent endonuclease, which mediates DNA fragmentation, is inhibited by zinc. However, this target is beyond the point of no return for programmed cell death, and an inhibition could explain a suppression of DNA fragmentation during apoptosis, but not the effect on cellular survival. Another important group of enzymes in apoptosis is cysteine-aspartic acid proteases, also known as caspases. Inactive pro-caspases first are activated by proteolytic cleavage and second form a signaling cascade to transduce initial apoptotic signals to effector enzymes mediating the organized cellular destruction characteristic for programmed cell death. Low micromolar zinc concentrations are reported to inhibit caspases-3, -6, and -8 [242]. A half maximal inhibitory concentration for caspase-3 was found below 10 nM [99]. This value is within the physiological range of free intracellular zinc level, leading to the suggestion that endogenous zinc can inhibit caspase-3 activity subsequently altering apoptosis signals and cellular survival. More recently, similar results were observed in an in vivo rat heterotrophic heart transplant model that might contribute to an increased allograft survival [87].

Even though both cell types of the adaptive immune system utilize the same signaling pathways, B cell function seems to be affected by zinc to a lesser magnitude. Even the reduced antibody production during zinc deficiency is based on reduced B cell numbers whereas it remains unaffected on a per-cell basis [243]. This indicates an effect on cellular development rather than on function. One reason might be a difference in zinc homeostasis, making mature B cells less susceptible to conditions of limited zinc availability. Although B cells are highly susceptible to apoptosis during development, and zinc is one factor that influences these signals, mature B cells can tolerate comparable conditions due to changes in zinc-regulating proteins, but also by changing the expression patterns of several other factors that regulate the cellular responsiveness to apoptotic signals. One highly important zinc-regulating protein in B cells is the zinc importer Zip10.

Zip10 is essential for cell survival during early B-cell development as well as for adequate B-cell receptor (BCR) signaling [244,245]. A loss of Zip10 during an early B cell stage was reported to specifically abrogated cell survival. Eventually, this leads to an absence of mature B cells in vivo, provoking spleno-atrophy and reduced Ig level. Moreover, the absence of Zip10 causes an impaired T cell-dependent and -independent immune response respectively. Additionally, due to the dysregulated BCR signaling, mature B cells proliferated poorly in response to BCR crosslinking. On the molecular level, this is probable due to a disturbed JAK-STAT-Zip10 signaling axis, since the JAK-STAT pathway is known to modulate expression of Zip10 [244]. Furthermore, a disturbed regulation of the BCR signal strength is likely. This might be due to the positive regulator function of Zip10 regarding CD45R phosphatase activity. In the case of Zip10 malfunction, CD45R phosphatase activity is reduced resulting in hyperactivation of tyrosine-protein kinase Lyn that consequently provoke an altered threshold for BCR signaling [245].

## 7. Conclusions

Summing up, all immune cells are directly affected by zinc signals. Most prominent pathologic changes are found during zinc deficiency indicating that zinc is a main regulator of cellular function and signal transduction. However, precise underlying molecular mechanisms still need to be investigated in further detail, to guarantee beneficial effects of clinical zinc application to patients suffering of distinct diseases.

## Figures and Tables

**Table 1:** Effect of zinc signals on Immune function: Altered immunological functions can be induced by different zinc signals, as zinc flux, zinc wave, and homeostatic zinc signal. Altered from [59].

Zinc Signal	Duration	Effect
Zinc Flux	Seconds/minutes	▪ Inhibition of PDE in monocytes/macrophages [60] ▪ Inhibition of MKP in monocytes/macrophages [61,62] ▪ Induction of PMA-triggered NET-formation in PMN [40] ▪ Induction of Lck recruitment to TCR [63,64] ▪ Zinc release from lysosomes in T cells [65] ▪ Triggering T cell activation by APC [66] ▪ Induction of Lck homodimerization/activation in T cells [67] ▪ Redistribution of zinc from nucleus/mitochondria to cytosol/microsomes [30]
Zinc wave	Minutes	▪ Zinc release from perinuclear area in mast cells [57]
homeostatic Zinc Signal	Hours	▪ Stabilization of MyD88 expression [5,68] ▪ Induction of A20 expression in T cells, monocytes [69] ▪ Negative regulation of IRAK signaling [70,71] ▪ MAPK activation in immune cells [72,73,74] ▪ Negative regulation of TRIF pathway in macrophages [5] ▪ Changed Zip and ZnT expression in DCs and T cells [58,72] ▪ Inhibition of AC transcription in T cells [75] ▪ Influence of cytokine production, e.g., IL-2 in T cells [72] ▪ Induction of Akt/ERK/p38 phosphorylation in T cells [73] ▪ Triggering of PTEN degradation in T cells [76] ▪ Inhibition of IL-6/IL-1 induced STAT3 phosphorylation [77] ▪ Alteration of M1/M2 differentiation by inhibition of STAT6 phosphorylation [78] ▪ Induction/stabilization of regulatory T cells [79,80] ▪ Inhibition of Th17 and Th9 cell differentiation [81,82] ▪ Reduced cytokine production, e.g., IFN-γ in T cells [83] ▪ Induction of NK cell killing and granzyme expression [84,85] ▪ Inhibition of caspase activity ▪ Inhibition of cAMP/cGMP hydrolysis [86,87] ▪ Inhibition of DNA fragmentation [6] ▪ Epigenetic modifications due to inhibition of SIRT1 [79] ▪ Inhibition of IL-1/TNF CRP expression in myeloid cells [53,69,88]

AC: adenylate cyclase; cAMP: cyclic adenosine monophosphate; cGMP: cyclic guanine monophosphate; CRP: c-reactive protein; DC: dendritic cell; IL: interleukin; IFN: interferon; IRAK: interleukin-1 receptor-associated kinase; Lck: lymphocyte-specific protein tyrosine kinas; MAPK: mitogen-activated protein kinase; MKP: MAP-kinase phosphatase; MyD88: myeloid differentiation primary response gene 88; NET: neutrophil extracellular traps; NK: natural killer; PDE: phosphodiesterase; PMA: Phorbol-12-myristat-13-acetat; PTEN: phosphatase and tensin homolog deleted on chromosome 10; STAT: signal transducers and activators of transcription; TCR: T cell receptor; APC: Antigen presenting cell; Th: T helper; TNF: tumor necrosis factor; TRIF: Toll-interleukin-1 receptor (TIR) domain-containing adaptor-inducing interferon.

**Table 2:** IC_50_ values for signaling molecules in humans.

Signaling Molecule	IC_50_	Reference
PTPRB	98 pM	[114]
PTEN	0.6 nM	[76]
PTP1B	3–17 nM	[100]
SHP-1	92 nM	[115]
SHP-2	1–2 µM	[100]
TC PTP	200 mM	[99]
Calcineurin	250 nM–7 µM	[116]
Ca-dependent endonuclease	1 µM (in the presence of 10 µM Ca)	[117]
LAR	20 µM	[100]
PDE4A	>5 µM	[118]
PDE 5	>1 µM	[119]
PDE 6	>1 µM	[120]
IKK	8.7 µM	[121]
Caspase 3	<10 nM, 100 nM	[86,99]

PTPRB: Protein phosphotyrosylphosphatase receptor type B; PTEN: Phosphatase and tensin homologue deleted on chromosome 10; PTP1B: Protein phosphotyrosylphosphatase 1B; SHP: SH2-containing phosphatase; TC PTP: T cell PTP; LAR: Leukocyte common antigen-related protein; PDE: phosphodiesterase; IKK: IκB kinase.
